# Metabolites Profiling, In Vitro, In Vivo, Computational Pharmacokinetics and Biological Predictions of *Aloe perryi* Resins Methanolic Extract

**DOI:** 10.3390/plants10061106

**Published:** 2021-05-30

**Authors:** Rasha Saad Suliman, Sahar Saleh Alghamdi, Rizwan Ali, Dimah A. Aljatli, Sarah Huwaizi, Rania Suliman, Ghadeer M. Albadrani, Abdulellah Al Tolayyan, Bandar Alghanem

**Affiliations:** 1 College of Pharmacy, King Saud bin Abdulaziz University for Health Sciences, Riyadh 14611, Saudi Arabia; ghamdisa@ksau-hs.edu.sa (S.S.A.); aljatli097@ksau-hs.edu.sa (D.A.A.); 2 Medical Research Core Facility and Platforms, King Abdullah International Medical Research Center (KAIMRC), Ministry of National Guard Health Affairs, Riyadh 11481, Saudi Arabia; aliri@ngha.med.sa (R.A.); Huwaizisa@ngha.med.sa (S.H.); altolayyanab@ngha.med.sa (A.A.T.); ghanemba@ngha.med.sa (B.A.); 3 Clinical Laboratory Sciences, Prince Sultan Military College of Health Sciences, Dahran 34313, Saudi Arabia; wereru@hotmail.com; 4 Department of Biology, College of Science, Princess Nourah bint Abdulrahman University, Riyadh 11564, Saudi Arabia; gmalbadrani@pnu.edu.sa

**Keywords:** *Aloe perryi* resins methanolic extract, in vivo anti-inflammatory, leukemia cancer, breast cancer, ADME, target prediction

## Abstract

Background: *Aloe perryi* is a traditional herb that has various biological and pharmacological properties such as anti-inflammatory, laxative, antiviral, antidiabetic, and antitumor effects, which have not been deliberated before. The current investigation aims to evaluate in vitro cytotoxicity against several cancer cell lines in addition to in vivo anti-inflammatory activities of *Aloe perryi* extract using a rat animal model. Moreover, the pharmacokinetic properties of bioactive constituents and possible biological targets were assessed and evaluated. The methanolic extract of *Aloe perryi* was prepared by maceration, to tentatively identify the biomolecules of the *Aloe perryi* extract, analytical LC–QTOF-MS method was employed for *Aloe perryi* methanolic extract. The cytotoxic activity was examined in six cancer cell lines using Titer-Glo assay and the IC_50s_ were calculated in addition to in silico target predictions and in vivo anti-inflammatory activity assessment. Subsequently, the pharmacokinetics of the identified active components of *Aloe perryi* were predicted using SwissADME, and target prediction using the Molinspiration webserver. The cytotoxic activity on HL60 and MDA-MB-231 was moderately affected by the *Aloe perryi* extract with IC_50_ of 63.81, and 89.85 μg/mL, respectively, with no activity on other cells lines. Moreover, the *Aloe perryi* extract exhibited a significant increase in wound contraction, hair growth, and complete re-epithelization when compared with the negative control. The pharmacokinetic properties of the bioactive constituents suggested a good pharmaceutical profile for the active compounds and nuclear receptors and enzymes were the two main possible targets for these active compounds. Our results demonstrated the promising activity of *Aloe perryi* extract with cytotoxic and anti-inflammatory properties, indicating a potential therapeutic utility of this plant in various disease conditions.

## 1. Introduction

The discovery of medications and identification of therapies began with an experimental approach to treat diseases based on the pharmacological or biological activities of natural products [1,2]. Natural products and their analogs have played a significant role in human health where they have shown potential activities such as anticancer, antimicrobial, and anti-inflammatory [3,4]. Furthermore, medicinal sources can be widely classified into four classes, one of which is plants, which is a primary source of natural products, such as paclitaxel, that is derived from *Taxus brevifolia* [5,6]. Moreover, plant-derived compounds have shown great promise as clinically useful cytotoxic medications. According to Cragg et al., vinblastine, vincristine, camptothecin derivatives, topotecan, irinotecan, and etoposide are derived from *epipodophyllotoxin* [7]. In addition, naturally derived compounds are favorable for the treatment of cancer due to their anti-proliferative activity, safety, availability, and the possibility to overcome the resistance [8,9]. Cancer remains the second and biggest driving reason for death worldwide, and it is defined as a genetic disease that starts with the abnormal and unregulated division of ordinary cells in the body that generates a mass of cells called a tumor, which acquire a metastatic potential to other organs [10,11]. Cancer can be classified as carcinoma, leukemia, sarcoma, lymphomas, and mixed type [12]. Although the survival rate is increasing because of early detection, the National Cancer Institute reported an estimation of 1,806,590 new cancer cases diagnosed in the US and 606,520 deaths in 2020. Moreover, among all cancer types, breast cancer, lung cancer, prostate cancer, colorectal cancer, melanoma, and bladder cancer are considered to be the most common cancers with an estimated 43% of all cases are prostate, lung, and colorectal cancers. It is considered a complex disease that occurs for multiple reasons, one of these is DNA damage caused by environmental agents, ionizing radiations, and ultraviolet radiations [8]. Until present, the effective mechanism for treating cancer has not yet been fully established, [13] whereas most of the currently used cancer treatments include a combination of chemotherapy, radiotherapy, surgeries, or hormonal therapy [14]. Despite its long clinical success and the availability of multiple anticancer agents, there are several limitations, such as multi-drug resistance, solubility, adverse side effects, and poor bioavailability, which demonstrates that cancer treatment remains an unmet need and requires the development of new therapies [15]. These circumstances force scientists to investigate new cytotoxic agents; over the last two decades, many laboratories have begun to study natural products and their anticancer activity on humans.

Nowadays, natural products, specifically plants, have provided huge opportunities in drug development because of their chemical diversity [16]. *Aloe* species are among the most widely used plants and are considered to be one of the traditional medicines in many countries due to their therapeutic properties. In Africa, there are more than 500 species of *Aloe* belonging to the *Aloeaceae* family, which broadly grow in tropical and subtropical regions [17]. *Aloe Vera*, *Aloe perryi*, *Aloe arborescens*, and *Aloe ferox* are the most widely recognized and most broadly used of all Aloe species [18]. The main differences between *A. Perryi* and *A. Vera* species are that *A. Vera* has yellow flowers, while *A. perryi* has red flowers [19]. Moreover, *Aloe perryi* is a perennial plant, typically reaching 30 cm tall, which often grows in rocky areas [18]. *Aloe* leaves contain many classes of bioactive compounds including chromones, flavonoids, anthraquinones, anthrones, amino acids, lipids, carbohydrates, vitamins, and minerals [20]. Furthermore, the unique compound of *Aloe*, which has not been found in any other plant, is C-glycosylated chromones, which are considered as a phenolic compound that possesses antioxidant, antimicrobial, anticancer, and anti-inflammatory activities [21]. On the therapeutic level, many studies reported the activity of *A. perryi* in the treatment of eye [19]., constipation, malaria [22], antioxidant, antidiabetic, anti-tumor, anti-inflammatory, wound-healing properties and antimicrobial activity [20].

In this regard, Al-Oqail et al. investigated the anti-proliferative activity of *Aloe perryi* flower extract against liver, colon, breast, lung, prostate, and epithelial human cancer cell lines using petroleum ether, chloroform, ethyl acetate, butanol, and aqueous extracts. The authors have shown that different extracts of *Aloe perryi* have significant cytotoxic activity against various cancer cell lines. As shown in Table 1 below, petroleum extract was the most active extract among all the above-mentioned extracts. On the contrary, HCT-116 were the most sensitive cells with an IC_50_ of 5.61 μg/mL. The authors also confirmed the existence of a variety of phytochemicals, namely glycosides, phenols, proteins, flavonoids, and phytosterols. In conclusion, these results suggest a potential anti-proliferative activity of *Aloe perryi* and could be a promising cytotoxic agent [16].

Furthermore, the development of new anti-inflammatory compounds has gained great interest. Topical products of *Aloe* are used for their anti-inflammatory and wound healing properties for acne, abrasions, sunburn, minor burns, diabetic ulcers, and stomatitis. It works by inactivation of bradykinin to inhibit PGA2, arachidonic acid oxidization, and thromboxane A blocking. *Aloe* activity may result from its ability to increase blood flow in the affected area [23]. Unfortunately, *Aloe perryi*’s anti-inflammatory activity remains poorly investigated. Until present, there have been few to no reported studies that have assessed the anti-inflammatory properties of *Aloe perryi* resins. Therefore, we sought to determine the cytotoxic, anti-inflammatory properties of *Aloe perryi* resins in addition to evaluating the pharmacokinetic properties of the active compounds with predicted molecular targets that could potentially mediate these biological effects.

## 2. Results

### 2.1. Botanical Material Sampling and Extract Preparation

The supernatant layer was collected and concentrated to 100 mL using a rotary evaporator. The weight of the discarded filtrate was 80 gm. Therefore, the final concentration of the extract was 70 gm in 100 mL.

### 2.2. Metabolites Profiling Using HPLC and QTOF/LCMS

The methanolic extract of the *Aloe perryi* was evaporated to dryness, then was subjected to analysis by the analytical RP-HPLC method. The obtained extracts were subjected to HPLC–DAD followed by LC/MS.

Chemicals of formic acid and HPLC grade Methanol were purchased from Sigma-Aldrich (St. Louis, MO, USA) and Honeywell (Paris, France), respectively. The methanolic extract was first injected into the Agilent 1260 Infinity HPLC system (AGILENT, Waldbronn, Germany) with Diode-Array Detection (DAD) detector. The separation carried out in a reversed-phase mode using Phenomenex Kinetex-C18 column (4.6 mm × 250 mm, 5 μm) with the following elution gradient; 0–1 min, 5% B; 1–11 min, 5–100% B; 11–13 min, 95%B; 13–15 min, 5%B; 15–16 min, 5%B using mobile phase A (0.1% HCOOH in water) and mobile phase B (0.1% HCOOH in Methanol). Samples were injected with 20 μm injection volume and the flow rate was set as 1 mL/min. The DAD collected UV spectrum at 200 nm, 225 nm, 250 nm, 275 nm, 300 nm, 325 nm, 350 nm, and 400 nm (nanometers). The data were processed using Chemstation software.

**Figure 1:**
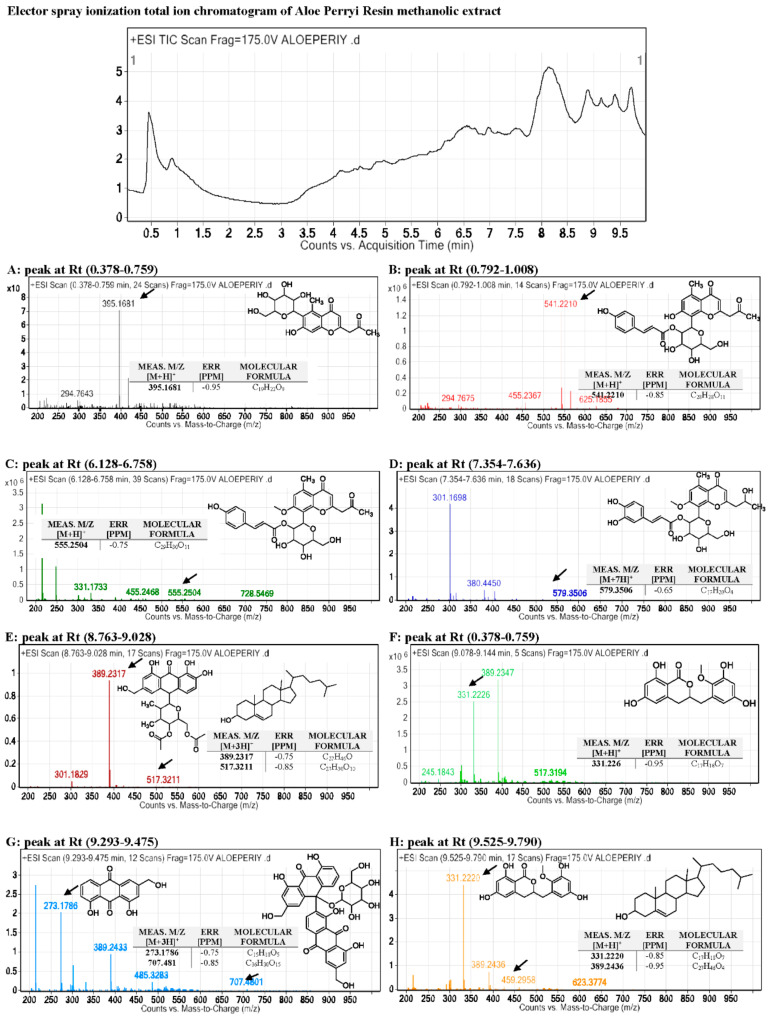
Base peak chromatogram of *Aloe perryi* crude extract and tentatively identified secondary metabolites, which are Isoaloesin (**A**), Nataloe emodin-8-methyl ester (**B**), 7−O−Methylaloeresin A (**C**), Isorabaichromone (**D**), 7−hydroxyaloin−4′,6′−O−diacetate and cholesterol (**E**), Aloe coumarin (**F**), Aloe emodin and glycosylated dimer derivative elgonicardine (**G**), Aloe coumarin and cholesterol (**H**) indicates that *m*/*z* implies measured *m*/*z*.

**Figure 2:**
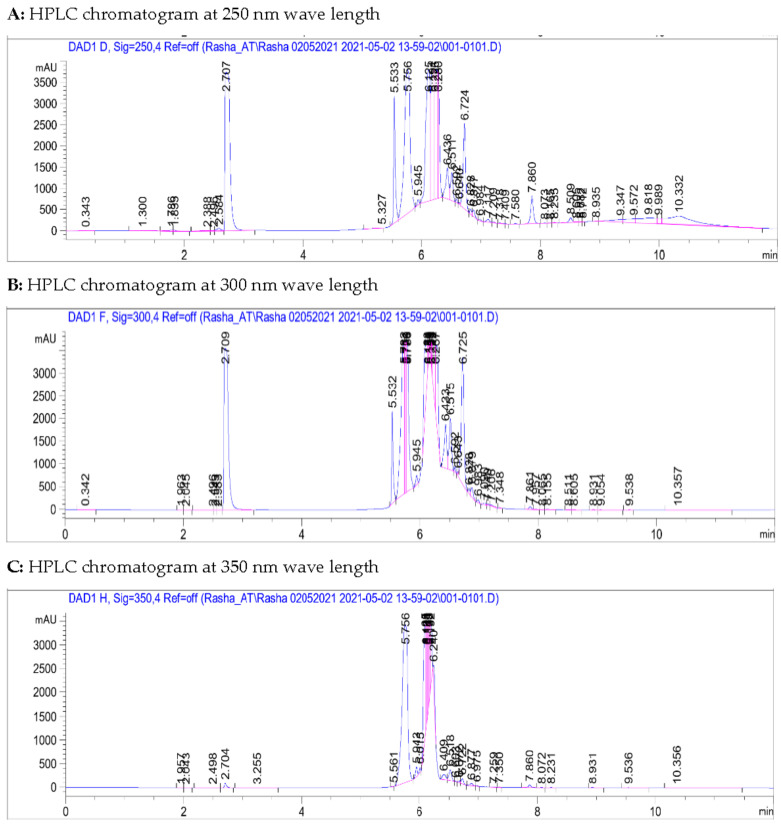
HPLC Chromatograms of the *Aloe perryi* Resins Methanolic Extract at Different Wavelengths (**A**–**C**). It is demonstrated from the first chart at wave length 250 nm (**A**), that there is a single peak eluted at 2.7 min retention time, followed by a bundle of around nine (9) peaks eluted in the area of 5.5 to 7.8 min retention time. The second wave length at 300 nm (**B**) showed almost the similar elution, with one peak that not eluted at 7.8 min in the area of non-polar compounds. Lastly, there is only a group of a non-polar compounds appears within the retention time of 5.5–6.4 min retention time at wave length 350 (**C**). It could be concluded that 250 nm represent the best elution and separations for the peaks. nm: nanometers; mAU: milli Absorbance Unit; Em: emission; Ex: excitation.

On the other hand, the total ion current spectra (TIC) raw data was obtained using the qualitative and quantitative data-analysis program Mass Hunter (Agilent Technologies). After conducting a mass screening on the below spectrum, we summarized the following: chemical features were extracted from the LC-MS data using the molecular features extraction (MFE) algorithm and the recursive analysis workflow. Features have been extracted by screening the detected nodes at various retention times per minute, with a minimum intensity of 6,000 counts and aligned with previously detected compounds considering adducts ([M+H]^+^, [M+2H]^+^, [M+Na]^+^ and [M+4H]^+^). Those compounds are tentatively identified as follows: Isoaloesin, 7−O−methylaloeresin A, Isorabaichromone, 7−hydroxyaloin-4′,6′−O−diacetate, Cholesterol, Aloe coumarin, Aloe emodin, and glycosylated dimer derivative elgonicardine. All of the isolated and identified compounds are phenolic phytochemicals with several subclasses. The identified classes including three (3) anthraquinones, two (2) chromones, two (2) anthrones, two (2) Coumarin, and two (2) sterols (see Figure 1 and Figure 2).

### 2.3. Treatment with Aloe perryi Extract Demonstrates Cytotoxic Properties against Several Cancer Cells

**Figure 3:**
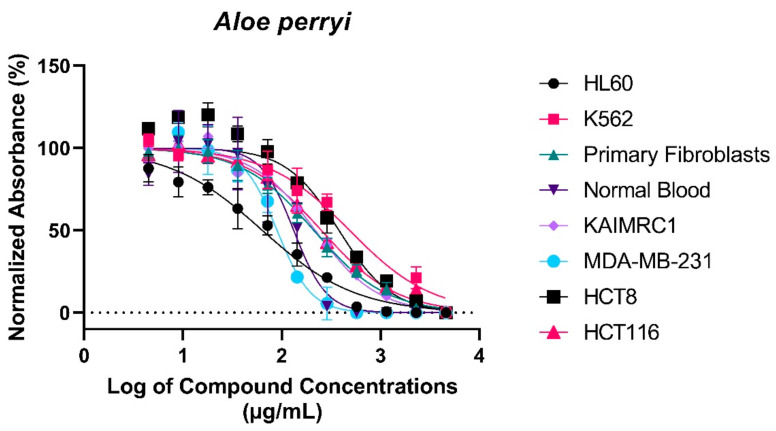
Graphical Representation of dose–response curve of *Aloe perryi* extract.

In this experiment, we sought to determine the cytotoxic properties of Aloe perryi extracts against several cancer cell lines. Therefore, the MTT assay was used to perform the experiment. Post *Aloe perryi* treatment, the viability of breast cancer cell lines, MDA MB-231 and KAIMRC1, colorectal cancer cell lines, HCT8 and HCT116, Leukemia Cell lines, HL60, and K562, and normal primary fibroblasts and normal blood cells were determined. A dose–response curve for the half-maximal inhibitory concentration IC_50_ (µg/mL) of *Aloe perryi* extract was plotted (Figure 3). The cytotoxicity effect at a concentration <10 μg/mL is considered strongly active, and from 11–100 μg/mL is considered moderately active, whereas above 100 µg/mL is considered non-active. Among all the tested cell lines, the viability of Acute Myeloid Leukemia (AML), HL60 cell line (63.81 μg/mL) and breast cancer MDA-MB-231 cell line (89.85 μg/mL) were moderately affected by the *Aloe perryi* extract. The extract showed no activity on other cell lines, including the normal cells, suggesting selectivity towards cancer cells only (the IC_50_s are summarized in Table 2).

Several primary and cancer cell lines were treated with *Aloe perryi* extract. Cell viability was assessed using TiterGlo™ assay. IC_50_ values were calculated using an eleven-point serial dilution. The X-axis represents percent normalized absorbance and the y-axis represents log of *Aloe perryi* concentrations in µg/mL.

#### 2.3.1. Prediction of Biological Activity Using PASS Online

Analysis of *Aloe perryi’s* methanolic extract was incorporated using the online service prediction of activity spectra for substances (PASS) in order to determine the biological probability of a substance being active (Pa) or inactive (Pi) [24]. As shown in Table 3, between the 10 compounds, compound 2 was revealed to be the most promising compound as an anticarcinogenic and antineoplastic with Pa values of 0.820 and 0.814, respectively. These results further confirm the in vitro results that *Aloe perryi* methanolic extract possesses a cytotoxic activity that could be further investigated.

#### 2.3.2. Topical Treatment with *Aloe perryi* Extract Increases Wound Closure in the Skin of Healthy Rats

**Figure 4:**
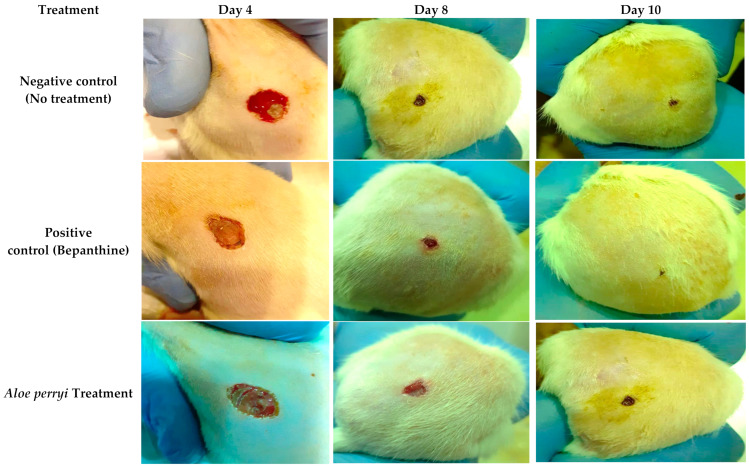
Representation of in vivo testing of the anti-inflammatory activity of negative control, Bepanthine, and *Aloe perryi* treatment. *Aloe perryi* and Bepanthine improved wound contraction, hair growth, and the proliferative stage maturation of the blood vessels.

To evaluate the anti-inflammatory activity of *Aloe perryi* extract, a skin wound was performed on the rat skin and the wound was evaluated on 4, 8, and 10 days with negative (no treatment), positive (Bepanthine) controls and *Aloe perryi* extract treatment. As shown in Figure 3, all rats were on the stage when the blood clots formed, the bleeding stopped, and fibrin formed. Our results demonstrate that Bepanthine treatment exhibited the highest contraction in comparison to the control and *Aloe perryi* treatment. One possible explanation is that Bepanthine contains Dexapanthol which penetrates the inner layers of the skin and moisturizes, heals, and regenerates the skin. Moreover, Dexapanthol helps to oxygenate the skin that is important in the healing process due to blood and oxygen availability on many metabolic processes. The application of *Aloe perryi* treatment resulted in wound contraction that is comparable to the negative control. It should be noted that the wound contraction of *Aloe perryi* and negative control was similar in size; however, the histological assessment of both tissues suggested a major difference in the skin layers and tissues confirming the anti-inflammatory effect of *Aloe perryi* treatment. Additionally, *Aloe perryi* treatment enhanced wound contraction, hair growth, and proliferative stage maturation of the blood vessels which was also observed with Bepanthine treatment, as represented in Figure 4.

### 2.4. Topical Treatment with Aloe perryi Extract Enhances the Complete Re-Epithelialization in the Skin of Healthy Rats

**Figure 5:**
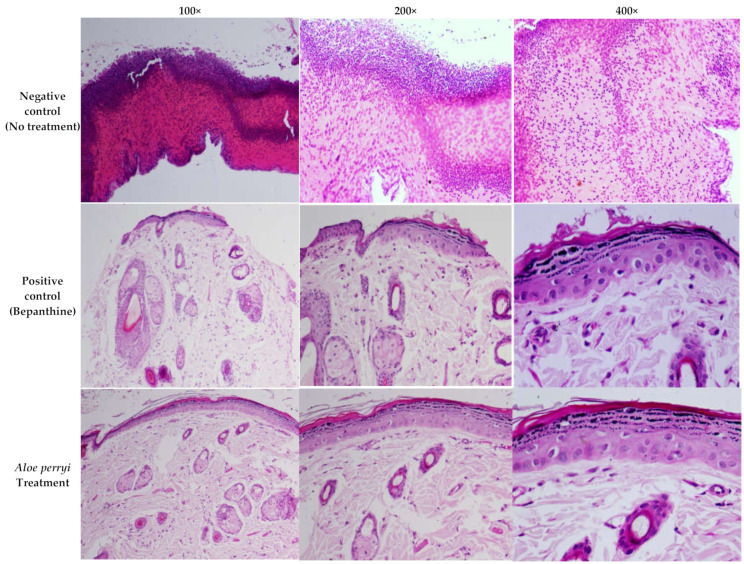
The histological changes in rat skin wounds treated with negative control, Bepanthine, and *Aloe perryi* extract. Bepanthine, and *Aloe perryi* treatment demonstrated a complete re-epithelialization, thickening of collagen fibers, and reducing the number of inflammation cells comparable to the negative control group.

Histological changes in the wound are a major area of interest during the wound-healing process, as presented in Figure 5. The negative control showed diffused bleeding and inflammatory cells, particularly in neutrophils within the loose connective tissue, with the presence of immature granulation tissue revealing massive tissue injured in epithelial, connective, and subcutaneous tissues, accompanied by severe inflammatory cell diffusion, and inflammatory cells in scar tissue. On contrary, Bepanthine, as a positive control, showed normal skin tissues with mild keratinized tissue removal. Moreover, the layer of the epidermis grew thicker with granular tissue that showed cells and arteries filled out the wound. Treatment with *Aloe perryi* extract resulted in a complete re-epithelialization and a normal epidermis covered the wound area, and collagen fibers were thicker and denser with some cutaneous annexes, such as sebaceous glands and hair follicles in the center of the scar. In addition to the number of inflammation cells, which were less than the negative control group.

### 2.5. Pharmacokinetics ADME Predictions of the Bioactive Constituents of Aloe perryi Extract

Using the SwissADME webserver, the Computational ADME properties were determined for the *Aloe perryi* active compounds as summarized in Table 4.

#### 2.5.1. Molecular Weight (MW)

According to Lipinski’s rule which limits molecular weight (MW) to less than 500, the active compounds of *Aloe perryi* extract appears to follow the rule for most of the compounds, except for compounds 2, 3, 5, and 8 with molecular weights of 540.52, 554.54, 514.52, 702.61 g/mol, respectively [25].

#### 2.5.2. Lipophilicity (Log P) and Gastrointestinal (GI) Absorption

Log P represents the compound lipophilicity, according to SwissADME software, the recommended range should be within (-2.06.5) in order to be accepted and exhibit good oral absorption. All Aloe perryi compounds were within the recommended range, except for compounds 4 and 10 with log P values of 7.39 for both, which were above the recommended values. In addition to that, all Aloe perryi compounds were not good candidates for GI absorption, except for compounds 6, 7, and 9 [25,26].

#### 2.5.3. Solubility (Log S)

Log S is the aqueous solubility of compounds that directly influence oral absorption. All compounds showed soluble to moderate solubility within the recommended range (−6.5–0.6) [25].

#### 2.5.4. Blood–Brain Barrier (BBB) Permeability

All Aloe perryi compounds are unable to cross the blood–brain barrier (BBB) which could be a good feature since it will not exhibit CNS side effects. Correspondingly, the medicinal modification to the active constituents could be helpful to increase blood–brain barrier permeability, which suggests useful application in CNS tumors.

#### 2.5.5. Hydrogen Bond Donor and Acceptor (HBD/HBA)

The hydrogen bond donors (HBD) increase acidity, while the hydrogen bond acceptor (HBA) increases basicity. According to SwissADME, all compounds have an optimum number of HBD (0–5) except for compound 8, which has 10 hydrogen bond donors. Moreover, compounds 2, 3, and 8 exhibit the highest number of hydrogen bond acceptors, with 11, 11, and 15, respectively, which is considered a violation of the drug likeliness properties [25,27].

### 2.6. Target Prediction of the Bioactive Constituents of Aloe perryi Extract

As shown in Table 5, compounds with bioavailability scores above 0.00 are considered potential bioactive compounds. In other words, the greater the bioavailability score, the greater the possibility of the compound being biologically active at that target. According to Molinspiration, the results indicate that compound 8 showed no activity at any target. Moreover, compounds 4–6, 9 and 10 showed potential activity at G-protein-coupled receptors. Furthermore, molecules 4, 5, 7, and 10 are active as ion channel modulators. On the contrary, only compound 7 was active as a kinase inhibitor, in addition to that, the nuclear receptor ligand was the potential target for all compounds, excluding compound 8. Notably, all compounds were moderately active as protease inhibitors except 4, 5, 7, and 10 which exhibit good biological activity. Eventually, all compounds were active as enzyme inhibitors excluding compound 8, as mentioned above. To conclude, compounds 4, 5, and 10 could target five mechanisms, which are the GPCR ligand, ion channel modulator, nuclear receptor ligand, protease inhibitor, and enzyme inhibitor.

## 3. Discussion

### 3.1. Metabolites Profiling Using HPLC and QTOF/LCMS

*Aloe perryi* resins methanolic extract screening in HPLC/DAD revealed that methanolic extract is rich in a range of polar compounds and non-polar compounds. Those compounds showed that different Milli absorbance values (mAbs) vary from 3000 to 3500 within the areas of 250, 300, 350, and 410 nanometers (nm) wavelength. Moreover, there is a group of moderately polar compounds appears within the retention time of 5.5–6.8 min, this cluster requires further fractionation and orthogonal separation for those moderate polar group of compounds which is beyond the scope of this project.

On the other hand, the QTOF/LCMS analysis revealed the following: the *m/z* values at retention time (0.378–0.759) were correlated with the parent compound Isoaloesin from anthraquinones phytochemical class [28] with *m/z* 395.1681 and a molecular formula of [C_19_H_22_O_9_]^+^, in positive ion mode and [M-H]^−^ with *m/z* 393 in negative mode, indicating that the compound has a molecular weight of 394.126 g mol^−1^. The *m/z* values at retention time (0.792–1.008) were correlated with the parent compound Nataloe emodin-8-methyl ester from anthraquinones phytochemical class [29] with *m/z* 541.2210 and a molecular formula of [C_28_H_28_O_11_]^+^, in positive ion mode and [M-H]^−^ with *m/z* 539 in negative mode, indicating that the compound has a molecular weight of 540 g mol^−1^. The *m/z* values at retention time (6.128-6.758) were correlated with the parent compound 7-O-methylaloeresin A from the chromone phytochemical subclass of phenolic compounds [29] with *m/z* 555.2504 and a molecular formula of [C_29_H_30_O_11_]^+^, in positive ion mode, and [M-H]^−^ with *m/z* 553 in negative mode, indicating that the compound has a molecular weight of 554 g mol^−1^.

The *m/z* values at retention time (7.354-7.636) were correlated with the parent compound Isorabaichromone from chromone phytochemical subclass of phenolic compounds [29] with *m/z* [M+7H]^+^ 579.3506 and a molecular formula of [C_29_H_32_O_12_]^+^, in positive ion mode and [M-H]^−^ with *m/z* 571 in negative mode, indicating that the compound has a molecular weight of 572 g mol^−1^.

The first node’s *m/z* values at retention time (8.763–9.028) were correlated with the parent compound 7-hydroxyaloin-4′,6′-O-diacetate from anthrones phytochemical class of phenolic compounds [30] with *m/z* 389.2317 and a molecular formula of [C_27_H_30_O_10_]^+^ in positive ion mode and [M-H]^−^ with *m/z* 385 in negative mode, indicating that the compound has a molecular weight of 386 g mol^−1^.

The second node’s *m/z* values at retention time (8.763–9.028) were correlated with the parent compound cholesterol from the sterol phytochemical subclass of phenolic compounds [31] with *m/z* 517.2504 and a molecular formula of [C_27_H_46_O]^+^, in positive ion mode and [M-H]^−^ with *m/z* 513 in negative mode, indicating that the compound has a molecular weight of 514 g mol^−1^.

The *m/z* values at retention time (0.378-0.759) were correlated with the parent compound *Aloe coumarin* [32] from Coumarin phytochemical subclass of phenolic compounds, with *m/z* 331.226 and a molecular formula of [C_17_H_18_O_7_]^+^ in positive ion mode and [M-H]^−^ with *m/z* 329 in negative mode, indicating that the compound has a molecular weight of 330 g mol^−1^. The first node’s *m/z* values at retention time (9.293–9.475) were correlated with the parent compound *Aloe emodin* from anthraquinone phytochemical class of phenolic compounds [29] with *m/z* 273.1786 and a molecular formula of [C_15_H_10_O_5_]^+^ in positive ion mode and [M-H]^−^ with *m/z* 269 in negative mode, indicating that the compound has a molecular weight of 270 g mol^−1^. The second node’s *m/z* values at retention time (9.293–9.475) were correlated with the parent compound glycosylated dimer derivative elgonicardine from anthrones phytochemical subclass of phenolic compounds [29] with *m/z* 707.4801 and a molecular formula of [C_36_H_30_O_15_]^+^, in positive ion mode and [M-H]^−^ with *m/z* 703 in negative mode, indicating that the compound has a molecular weight of 704 g mol^−1^.

The first node’s *m/z* values at retention time (9.525–9.790) were correlated with the parent compound *Aloe coumarin* [32] from Coumarin phytochemical subclass of phenolic compounds, with *m/z* 331.226 and a molecular formula of [C_17_H_18_O_7_]^+^, in positive ion mode and [M-H]^−^ with *m/z* 329 in negative mode, indicating that the compound has a molecular weight of 330 g mol^−1^.

The second node that appeared at *m/z* value at retention time (9.525–9.790) were correlated with the parent compound cholesterol from sterols phytochemical subclass of phenolic compounds [31] with *m/z* 517.2504 and a molecular formula of [C_27_H_46_O]^+^ in positive ion mode and [M-H]^−^ with *m/z* 513 in negative mode, indicating that the compound has a molecular weight of 514 g mol^−1^.

Finally, there is an important point regarding QTOF-LC/MS results that needs to be highlighted, which is that those compounds are determined based on the screening in positive mode and negative mode only, which is not able to decide if there are two isomers or two compounds that have different stereochemistry. The most accurate identification required further fractionation and separation, followed by analysis in HPLC using a chiral column in order to confirm the absolute determination of the configuration of the identified compounds which is not determined in this study.

### 3.2. Cytotoxic Properties of Aloe perryi Extracts against Six Cancer Cell Lines

MDA-MB-231 cells are triple -negative, highly aggressive, invasive, and resistant to drug treatment. TNBCCs (Triple-negative breast cancer cells) often express epidermal growth factor receptor (EGFR) and it is associated with poor prognosis [33,34]. Small molecule tyrosine kinase inhibitors (TKIs) and the monoclonal antibody Cetuximab against EGFR are used in combination with cytotoxic drugs to show the inhibition of growth of TNBCCs in vitro [35]. There are several ongoing clinical trials to inhibit EGFR in TNBCs [36]. Similarly, *Aloe perryi* extract can also be utilized in combination with TKIs to inhibit TNBCCs, since it demonstrated good activity against this type of cancer. Moreover, treatment with *Aloe perryi* extract against leukemia cells (HL60) showed high cytotoxic activity, suggesting promising cytotoxic properties of this extract against multiple cancer cells that warrant further investigations.

The PASS online web server was used to predict the biological activity of bioactive constituents present in *Aloe perryi* methanolic extract and confirm our experimental in vitro results. Compound 2 demonstrated the highest Pa activity as anticarcinogenic and antineoplastic. Additionally, other compounds were also active as anticarcinogenic and antineoplastic agents that suggest the cytotoxicity of the methanolic extract could have resulted from the existence of these compounds.

### 3.3. Anti-Inflammatory Properties of Aloe perryi Extract

Historically, *Aloe* species were used as a topical treatment to treat the skin as a wound-healing plant to reduce the inflamed skin in traditional medicine [37]. Healing wound processes can be subdivided into three stages, containing inflammation, removal of dead tissue, and the final stage is leukocyte infiltration, which contains the formation of fibrous tissue and epithelial regeneration [38]. Our results showed that *Aloe perryi* treatment increased wound contraction, which was reported in the literature [39]. *Aloe* arborescens has proven skin wound treatment ability as a topical product. The study results indicated that the wound closure rate in animal models demonstrated that the whole-leaf juice assisted the healing process and inhibited microbial growth with no visible side effects. Furthermore, three *Aloe* species demonstrated the ability to speed the healing of wounds in keratinocytes with minor toxicity on normal keratinocyte cells [40]. The anti-inflammatory properties could be due to the availability of different forms of polysaccharides in *Aloe* species with quantity dependent on the plant’s age. Additionally, *Aloe* products could help in the production of more collagen when applied topically on the skin [41].

### 3.4. Histological Assessment of Skin Wound after Aloe perryi Extract Treatment

*Aloe* plant contains various classes of bioactive constituents that could contribute to the anti-inflammatory properties, including flavonoids, anthrones, anthraquinones, chromones, amino acids, lipids, carbohydrates, vitamins, and minerals [20]. Our results demonstrated a complete re-epithelialization and cover of the wound area with the normal epidermis, thickening of collagen fibers in addition to decreasing the number of inflammation cells after *Aloe perryi* treatment, confirming the anti-inflammatory effect of *Aloe perryi* extract.

### 3.5. Pharmacokinetics ADME Properties

Pharmacokinetics ADME properties are a key aspect in drug development in addition to pharmacodynamics. Recent evidence showed that the enhancement of compound safety and efficacy could be achieved by controlling physicochemical properties, such as molecular weight and lipophilicity, which leads to therapeutic success [26]. Moreover, molecular weight (MW) can correlate well with solubility, cellular membrane permeability, and passive diffusion, since low molecular weight compounds are probably highly absorbed, diffused, and transported across membranes, as shown on compounds 6 and 7 with MWs of 332.30 and 270.24 g/mol, respectively. On the contrary, high molecular weight molecules result in low GI absorption, in addition to the inability to crossing cellular membranes as readily as low molecular weight compounds, as presented in compounds 2, 3, and 5, which have high molecular weight [42]. Furthermore, the aqueous solubility of molecules has a fundamental effect on ADME properties, for example, one of the most notable causes of low oral bioavailability is low solubility and low permeability [43]. Correspondingly, by introducing hydrogen bond acceptors, the solubility of the compound could be enhanced which will help in improving the bioavailability of some compounds [44].

### 3.6. Target Prediction

Molinspiration is a virtual screening webserver that was utilized to calculate the bioavailability scores of compounds at multiple targets, such as GPCR ligands, enzymes, nuclear receptors, and ion channels [45]. Herein, our results showed that one of the possible targets for *Aloe perryi* extract is enzymes and, also, nuclear receptors since the computational predictions demonstrated the highest bioavailability scores with these two targets. This could provide a great benefit because the nuclear receptor ligands have shown great promise and potential in hormonal-dependent types of cancers, such as breast cancer. Therefore, our results suggest that the bioactive constituent of *Aloe perryi* extracts could serve as a targeted therapy for estrogen receptor α positive breast cancer or androgen receptor prostate cancer, which warrant further research and investigations [46].

## 4. Materials and Methods

### 4.1. Botanical Material Sampling and Extract Preparation

The plant material was collected in October 2020 from a plant nursery in the Riyadh region (Saudi Arabia) with an official authentication certificate. Briefly, 150 g of the plant powder material was macerated in 500 mL of absolute methanol at room temperature for 48 h and re-extracted three times using a similar procedure. Methanolic extracts were pooled, filtered, and the solvent was removed at 60ºC in the incubator chamber. The dried extracts were stored until further use. The yield was calculated using the following formula:

Yield (%) = (W1 × 100)/W2

where W1 was the weight of extract after the evaporation of the solvent, and W2 was the dry weight of the sample.

### 4.2. Chemical Identification

The extract obtained above was subjected to LC/MS. Using the following protocol.

#### 4.2.1. Chemicals

Formic acid and HPLC grade methanol were purchased from Sigma-Aldrich (St. Louis, MO, USA) and Honeywell (France), respectively.

#### 4.2.2. LC–QTOF-MS Method

The analysis of all extracts was performed on Agilent1260 Infinity HPLC system (Agilent, Germany) coupled with Agilent 6530 Quadrupole Time of Flight (Agilent, Singapore). Separation was performed using Agilent Extend-C18 column (2.1 mm × 50 mm, 1.8 μm) with the following elution gradient; 0–1 min, 5% B; 1–11 min, 5–100% B; 11–13 min, 95% B; 13–15 min, 5% B; 15-16 min, 5% B using mobile phase A (0.1% HCOOH in water) and mobile phase B (0.1% HCOOH in Methanol). The injection volume was 10 µl and the flow rate was set at 300 µl/min. The acquisition method MS1 was achieved in positive mode with mass ranging from 100-900 *m/z*. The mass spectrometer parameters were set as follows: Gas Temperature = 300 ℃; Gas flow = 8 L/min; Nebulizer =35 psi; Sheath Gas Temperature = 350 ℃ and Sheath Gas flow rate = 11 L/min. MS1 data were generated by Agilent MassHunter qualitative analysis software.

### 4.3. Cell Viability Assay

HL60, K562, MDA-MB-231, HCT8, and HCT116 cell lines were purchased from ATCC, USA, except KAIMRC1, which was isolated, established [47,48] and characterized [49] in the core laboratory facility KAIMRC, Riyadh, Saudi Arabia. Primary fibroblasts and normal blood samples were collected after obtaining informed written consent from the donors according to the Institutional Review Board (IRB) of King Abdullah International Medical Research Center (KAIMRC), Riyadh, Saudi Arabia. All the adherent cancer cell lines, including primary fibroblasts, were maintained in advanced DMEM containing 10% FBS, 1% L-glutamine, and 1% *Penicillin–Streptomycin* antibiotics. Non-adherent leukemia cell lines and normal blood cells were maintained in advanced RPMI containing 10% FBS, 1% L-glutamine, and 1% *Penicillin–Streptomycin* antibiotics. Flat-bottom white 96-well plates were used to plate cells at a density of 5 × 10^3^ cells/well in a 100 μL growth medium. The extract was serially diluted in DMSO. Cells were treated in 100 μL cell culture media in a serial dilution of the extract, ranging from 5mg to 0.01 μg/100µL in triplicate. The DMSO concentration in cell culture media was always 1% *v*/*v*. Cells were also treated with 1 μL of DMSO in 100 μL media to account for the effect of DMSO on the cells. Cells were incubated for 48 h at 37 °C with 5% CO_2_. Cell Titer-Glo assay (Promega) was used to determine the cell viability according to the manufacturer’s recommendations and Luminescence was measured using the Envision plate reader (Perkin Elmer). Acquired readings were normalized to average DMSO controls and expressed as a relative percentage. Analysis of data was performed on Graphpad Prism 8.1 software to determine the half-maximal inhibitory concentration (IC_50_).

### 4.4. In Vivo Anti-Inflammatory Testing

This study included the use of male Wistar rats (8 weeks old; 220–250 g) from Experimental Animal Care Center, Pharmacy College, King Saud University. Animals were divided into four groups of nine animals each. An inhalation anesthetic (Diethyl-Ether) was used to anesthetize the rats, which were then depilated before wounding at the predetermined site. A wound was perpetrated by removing approximately 1 cm full thickness of the assigned area on the lower back above the tail. The treatment of animals involved the external application of various plant extracts. The assigned groups of animals included the negative control group (no treatment), the positive control group (Bepanthine ointment), and the *Aloe perryi* treatment group. All treatments were applied to corresponding groups once every two days for 13 days, beginning from the day of wounding.

### 4.5. Histology

#### 4.5.1. Tissue Processing

Following dissection, tissues were transferred immediately into histological grade formalin (10%) for 24h, then placed in histological cassettes and kept in 70% ethanol before processing. Cassettes were processed on a tissue processor using a routine protocol. Briefly, cassettes were dehydrated in a series of ethanol solutions, starting with 70% and completing with 100%. Cassettes were cleared from ethanol by using multiple changes of xylene, and finally infused with paraffin wax (protocol details shown in Table 6) and the paraffin blocks were stored at 4 °C until sectioning.

#### 4.5.2. Sectioning

The paraffin blocks were sectioned at 4µm using a microtome. Sections were placed in a water bath at 45 °C and, carefully, one section was placed onto superfrost^®^ plus glass slides. The slides were dried overnight in an oven at 37 °C. Before staining, the slides were heated for 20 min at 60 °C on a hot plate.

#### 4.5.3. Haematoxylin and Eosin (H&E) Staining

The slides were dewaxed in two changes of xylene for 5 min each and rehydrated in three changes of graded ethanol (100%, 90%, and 70%) for 2 min each. The slides were washed with running tap water for 5 min. Cell nuclei were stained with Meyer’s hematoxylin for 15 s. The slides were rinsed in running tap water for 1 min. Sections were immersed in Scott’s solution (sodium hydrogen carbonate (0.35%; w/v), magnesium sulfate (2%; w/v) in dH2O) for 1 min and then rinsed in tap water for 1 min. The cells were stained with eosin (Eosin Y-solution 0.5% aqueous) for 2 min and then rinsed in tap water for 1 min. The slides were dehydrated for 1 min in two changes of graded ethanol (70% and 90%) and 2 min in 100% ethanol. The slides were transferred in two changes of xylene for 2 min each, and then permanently mounted in DPX mounting medium. The slides were imaged using a light microscope.

### 4.6. Pharmacokinetics ADME and Target Predictions

To calculate the pharmacokinetic properties of *Aloe perryi* active constituents such as absorption, distribution, metabolism, and elimination (ADME), the SwissADME webserver was utilized to generate these predictions. The SMILES of each bioactive compound present in the extract are summarized in Table 7. The generated SMILES were used for ADME predictions followed by results analysis according to the reported recommended range for each property.

### 4.7. Target Predictions

In addition to the ADME properties, a target prediction webserver was utilized to calculate the bioactivity scores against several molecular targets (www.molinspiration.com, accessed on 30 April 2021). The higher the bioactivity score, the more probable the molecule would be active at that target (positive values are actives, whereas negative values are in actives).

## 5. Conclusions

*Aloe perryi* exhibits several biological responses, which was reported by various studies indicated the properties of *Aloe perryi* in the treatment of eye problems, malaria, and constipation, with antimicrobial, anticancer, antioxidant, antidiabetic, anti-inflammatory, and wound-healing properties. Herein, we demonstrate the promising activity of *Aloe perryi* with cytotoxic, anti-inflammatory, and wound healing properties, which could be promising sources of new agents to treat acute myeloid leukemia, breast cancer, and inflammatory-based diseases. Nevertheless, there is a lack of studies that focus on the efficacy of *Aloe perryi* biological and therapeutic properties, mechanisms, and toxicological profile, suggesting further investigations and research are required for *Aloe perryi*.

## Figures and Tables

**Table 1:** The reported cytotoxic activities of *Aloe perryi* extracts.

Extract Type	Cell Line	Activity IC_50_ (μg)
Petroleum Ether extract	HepG-2 HCT-116 MCF-7 A-549 PC-3 HEp-2 HeLa	8.2 5.61 7.88 7.54 9.51 10.1 5.83
Chloroform Extract	HepG-2 HCT-116 MCF-7 A-549 PC-3 HEp-2 HeLa	17.3 14.1 22.9 13.0 21.7 18.7 12.1
Ethyl Acetate Extract	HepG-2 HCT-116 MCF-7 A-549 PC-3 HEp-2 HeLa	18.5 19.8 >50 18.8 >50 29.1 >50
Butanol Extract	HepG-2 HCT-116 MCF-7 A-549 PC-3 HEp-2 HeLa	16.9 10.3 12.7 18.8 19.7 26.7 11.1
Aqueous Extract	HepG-2 HCT-116 MCF-7 A-549 PC-3 HEp-2 HeLa	21.5 17.5 27.4 22.6 29.4 40.1 >50

HepG-2: Human hepatocellular carcinoma, HCT-116: Human colon cancer, MCF-7: Human breast cancer, A-549: Human lung adenocarcinoma, PC-3: Human prostate cancer, HEp-2: Human epithelial carcinoma, and HeLa: Human cervical cancer.

**Table 2:** *Aloe perryi* IC_50_ values. Breast cancer, colorectal cancer, and leukemia cell lines, primary fibroblasts, and normal blood cells were treated with graded concentrations of *Aloe perryi*, and IC_50_s were calculated. The *Aloe perryi* extract was dissolved in DMSO. R2 is the coefficient of differentiation and Se is the Standard error.

Cell lines		Treatment with *Aloe perryi* Extract		
		IC_50_ (µg/mL)	Se	R^2^
Leukemia	HL60	63.81	6.896	0.960
	K562	486.9	8.587	0.951
Normal primary fibroblasts	P1	211.6	5.048	0.983
Normal blood sample	N1	132.5	12.30	0.931
Breast cancer	KAIMRC1	218.5	6.059	0.979
	MDA-MB-231	89.85	6.947	0.977
Colorectal cancer	HCT8	380.3	11.24	0.950
	HCT116	252.2	4.136	0.988

**Table 3:** The Prediction of Cytotoxic Activity of Aloe perryi Active Compounds.

Compound Number	Predicted Activity	Pa	Pi
**1**	Anticarcinogenic Antineoplastic	0.716 0.50	0.008 0.035
**2**	Anticarcinogenic Antineoplastic	0.820 0.814	0.005 0.010
**3**	Anticarcinogenic Antineoplastic	0.806 0.802	0.005 0.012
**4**	Anticarcinogenic Antineoplastic	0.569 0.474	0.014 0.079
**5**	Anticarcinogenic Antineoplastic	0.627 0.821	0.011 0.009
**6**	Anticarcinogenic Antineoplastic	0.382 0.666	0.034 0.032
**7**	Anticarcinogenic Antineoplastic	0.510 0.722	0.018 0.022
**8**	Anticarcinogenic Antineoplastic	0.785 0.770	0.006 0.016
**9**	Anticarcinogenic Antineoplastic	0.382 0.666	0.034 0.032
**10**	Anticarcinogenic Antineoplastic	0.569 0.474	0.014 0.079

Pa: Probability of being active. Pi: Probability of being inactive.

**Table 4:** The pharmacokinetic ADME properties of the active constituents of *Aloe perryi* extract.

Compound Codes	Molecular Weight	HB Donor	HB Acceptor	Log Po/w (WLOGP)	Log S (SILICO S-IT)	BBB Permeability	GI Absorption	Rule of Five (ROF).
*Aloe perryi* Active Constituents								
**1**	394.37 g/mol	5	9	−0.87	−2.17 soluble	No	Low	Yes; 0 violation
**2**	540.52 g/mol	5	11	0.99	−4.32 Moderately soluble	No	Low	No; 2 violations: MW > 500, NorO > 10
**3**	554.54 g/mol	4	11	1.29	−5.00 Moderately soluble	No	Low	No; 2 violations: MW > 500, NorO > 10
**4**	386.65 g/mol	1	1	7.39	−5.78 Moderately soluble	No	Low	Yes; 0 violation
**5**	514.52 g/mol	4	10	2.35	−4.34 Moderately soluble	No	Low	Yes; 1 violation: MW > 500
**6**	332.30 g/mol	4	7	1.84	−3.35 Soluble	No	High	Yes; 0 violation
**7**	270.24 g/mol	3	5	1.21	−3.92 Soluble	No	High	Yes; 0 violation
**8**	702.61 g/mol	10	15	−0.49	−5.44 Moderately soluble	No	Low	No; 3 violations: MW > 500, NorO > 10, NHorOH > 5
**9**	332.30 g/mol	4	7	1.84	−3.35 soluble	No	High	Yes; 0 violation
**10**	386.65 g/mol	1	1	7.39	−5.78 Moderately soluble	No	Low	Yes; 0 violation

Recommended Ranges are: MW: <500 g/mol, HBD: <5, HBA: <10, Log P: −2.0–6.5, Log S: −6.5–0.6.

**Table 5:** Target predictions of the active constituents in *Aloe perryi* extract.

Compounds Codes	GPCR Ligand	Ion Channel Modulator	Kinase Inhibitor	Nuclear Receptor Ligand	Protease Inhibitor	Enzyme Inhibitor
	*Aloe perryi* Bioactive Constituents					
**1**	−0.16	−0.44	−0.40	0.00	−0.18	0.31
**2**	−0.09	−0.56	−0.34	0.08	−0.16	0.21
**3**	−0.11	−0.64	−0.38	0.02	−0.19	0.15
**4**	0.13	0.02	−0.51	0.75	0.04	0.54
**5**	0.13	0.04	−0.03	0.35	0.06	0.35
**6**	0.03	−0.10	−0.08	0.26	−0.12	0.06
**7**	−0.02	0.02	0.12	0.24	0.04	0.38
**8**	−0.71	−1.64	−1.05	−1.02	−0.29	−0.63
**9**	0.03	−0.10	−0.08	0.26	−0.12	0.06
**10**	0.13	0.02	−0.51	0.75	0.04	0.54

**Table 6:** The tissue processor: routine tissue processing protocol.

Reagents	Duration (Time)	Temperature (°C)
70% ethanol	30 min	37 °C
80% ethanol	30 min	37 °C
90% ethanol	30 min	37 °C
95% ethanol	30 min	37 °C
100% ethanol	1:00 h	37 °C
100% ethanol	1:00 h	37 °C
100% ethanol	1:30 h	37 °C
xylene	1:00 h	37 °C
xylene	1:30 h	37 °C
xylene	1:30 h	37 °C
paraffin wax	1:00 h	65 °C
paraffin wax	1:00 h	65 °C
paraffin wax	1:00 h	65 °C

**Table 7:** The generated SMILES for the active constituents in *Aloe perryi* extract.

SMILES	
**Compound 1**	CC(=O)CC1=CC(=O)C2=C(C)C(C3OC(CO)C(O)C(O)C3O)=C(O)C=C2O1
**Compound 2**	CC(=O)CC1=CC(=O)C2=C(C)C=C(O)C(C3OC(CO)C(O)C(O)C3OC(=O)\C=C\C3=CC=C(O)C=C3)=C2O1
**Compound 3**	COC1=CC(C)=C2C(=O)C=C(CC(C)=O)OC2=C1C1OC(CO)C(O)C(O)C1OC(=O)\C=C\C1=CC=C(O)C=C1
**Compound 4**	CC(C)CCCC(C)C1CCC2C3CC=C4CC(O)CCC4(C)C3CCC12C
**Compound 5**	CC1C(C)C(OC(COC(C)=O)C1OC(C)=O)C1C2=C(C(O)=C(O)C=C2)C(=O)C2=C1C=C(CO)C=C2O
**Compound 6**	COC1=C(O)C=C(O)C=C1CC1CC2=CC(O)=CC(O)=C2C(=O)O1
**Compound 7**	OCC1=CC(O)=C2C(=O)C3=C(O)C=CC=C3C(=O)C2=C1
**Compound 8**	OCC1OC(OC2(C3=CC=C4C(=O)C5=C(C(O)=CC(CO)=C5)C(=O)C4=C3O)C3=C(C(O)=CC=C3)C(=O)C3=C2C=C(CO)C=C3O)C(O)C(O)C1O
**Compound 9**	COC1=C(O)C=C(O)C=C1CC1CC2=CC(O)=CC(O)=C2C(=O)O1
**Compound 10**	CC(C)CCCC(C)C1CCC2C3CC=C4CC(O)CCC4(C)C3CCC12C

SMILES: Simplified molecular-input line-entry system.

## Data Availability

Not applicable.
